# Unusual Phenotype and Disease Trajectory in Kearns–Sayre Syndrome

**DOI:** 10.1155/2020/7368527

**Published:** 2020-02-27

**Authors:** Josef Finsterer, Michael Winklehner, Claudia Stöllberger, Thomas Hummel

**Affiliations:** ^1^Krankenanstalt Rudolfstiftung, Messerli Institute, Vienna, Austria; ^2^Institute of Neurology, Medical University of Vienna, Vienna, Austria; ^3^2nd Medical Department, Krankenanstalt Rudolfstiftung, Vienna, Austria; ^4^Krankenhaus Stockerau, Stockerau, Austria

## Abstract

**Objective:**

To describe unusual course and unusual phenotypic features in an adult patient with Kearns–Sayre syndrome (KSS). *Case Report*. The patient is a 49-year-old male with KSS, diagnosed clinically upon the core features, namely, onset before the age 20 of years, pigmentary retinopathy, and ophthalmoparesis, and the complementary features, namely, elevated CSF protein, cardiac conduction defects, and cerebellar ataxia. The patient presented also with other previously described features, such as diabetes, short stature, white matter lesions, hypoacusis, migraine, hepatopathy, steatosis hepatis, hypocorticism (hyponatremia), and cataract. Unusual features the patient presented with were congenital anisocoria, severe caries, liver cysts, pituitary enlargement, desquamation of hands and feet, bone chondroma, aortic ectasia, dermoidal cyst, and sinusoidal polyposis. The course was untypical since most of the core phenotypic features developed not earlier than in adulthood.

**Conclusions:**

KSS is a multisystem disease, but the number of tissues affected is higher than so far anticipated. KSS should be considered even if core features develop not earlier than in adulthood and if unusual features accompany the presentation.

## 1. Introduction

Kearns–Sayre syndrome (KSS) is a mitochondrial disorder (MID) most frequently due to a single mtDNA deletion and rarely due to mtDNA point mutations [1, 2]. The diagnosis is established clinically if the three core features onset, <20 years of age, chronic progressive external ophthalmoplegia (CPEO), and pigmentary retinopathy, and at least one of the following features are present: cerebrospinal fluid (CSF) protein >100 mg/dl, cardiac conduction defects, or cerebellar dysfunction [1]. Here, we present an adult male with KSS with unusual phenotypic features and disease trajectory.

## 2. Case Report

The patient is a 49-year-old male, with height 161 cm, weight 85 kg, and a history of preterm birth at gestational week 34 from nonconsanguineous parents, congenital anisocoria, developmental delay, hypoacusis, and tetra-ataxia (Table 1). During childhood general downslowing, chronic fatigue, and sicca syndrome of the eyes occurred. He repeatedly experienced respiratory infections and oral herpetic infections and once painless desquamation of the hands and foot soles (Table 1). He was successfully trained and worked as a hairdresser until the age of 38 years in his parent's hair saloon and retired at the age of 40 years.

**Figure 1:**
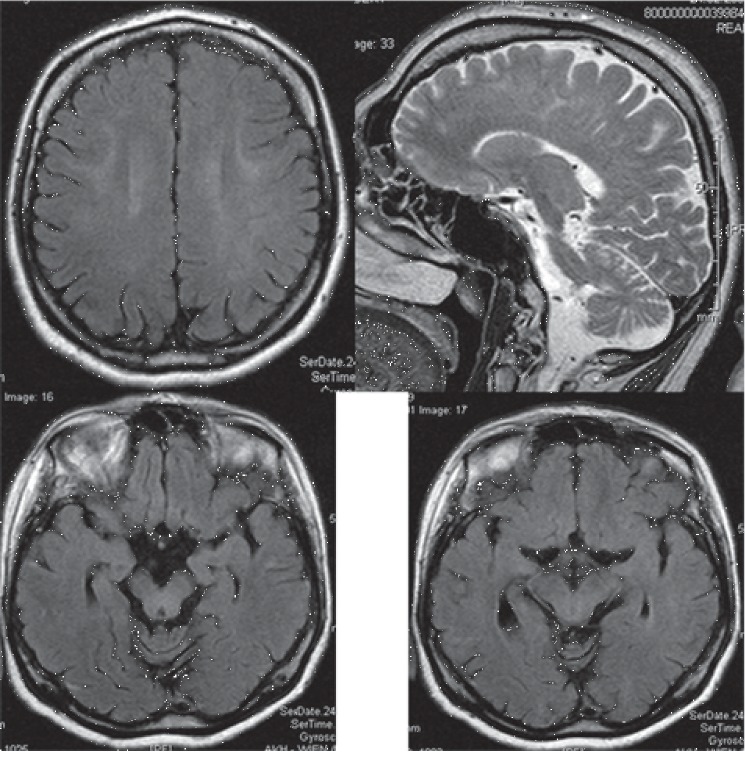
Cerebral MRI at age 36 showing mild, band-like T2-hyperintensities in the white matter bilaterally (upper left) and the midbrain (lower panels) and mild cerebellar atrophy (upper right).

**Figure 2:**
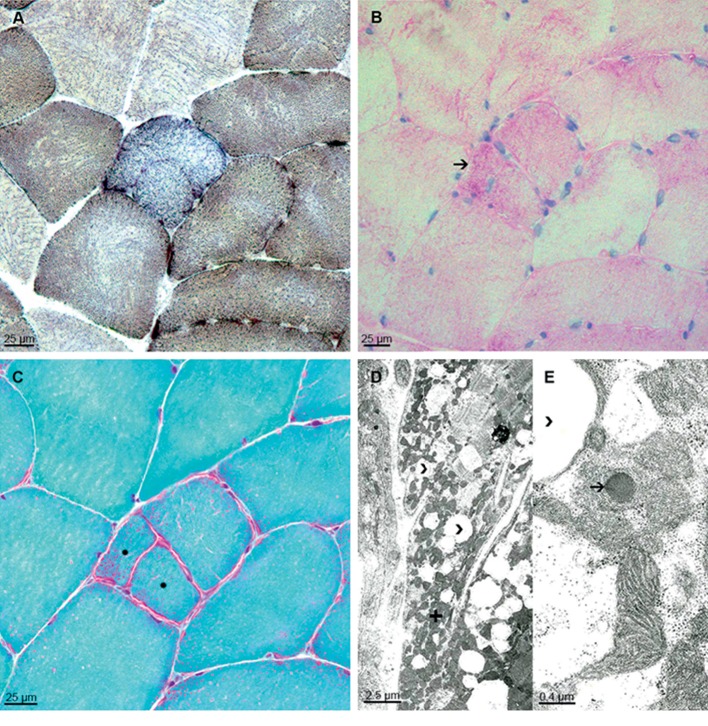
(a–c) Neuropathology of muscle biopsy (left vastus lateralis muscle) presents myopathic changes with single COX-negative fibers (a) COX/SDH double-labeling, mildly increased PAS-positivity in few fibers (b) arrow, muscle fiber splitting, and ragged-red fibers (c) Gomori-trichrome stain, asterisk. (d and e) Electron microscopy images illustrate increased numbers of fat vacuoles (d and e, arrow heads) between densly packed subsarcolemmal mitochondria (d) plus. Some mitochondria show single dark bodies in higher magnification (e) arrow.

**Figure 3:**
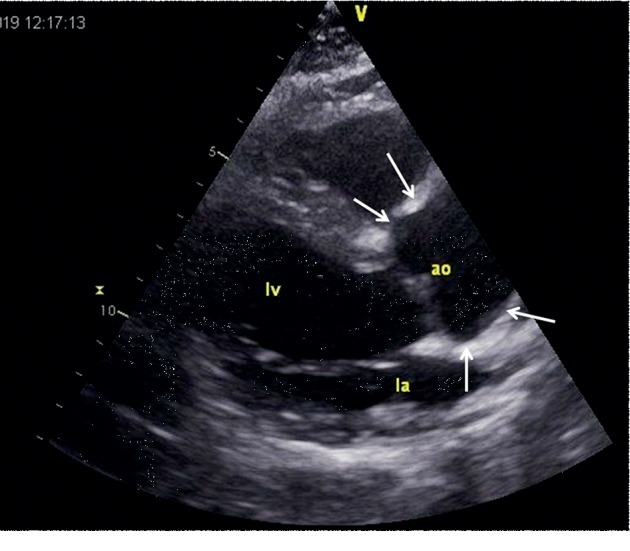
Echocardiographic parasternal long axis view showing the ectatic ascending aorta, measuring 37 mm in diameter.

At age of 30 years, left anterior hemiblock and right bundle-branch-block were recorded. Since age 35, recurrent mild elevation of creatine-kinase (CK) was noted. Aldolase and myoglobin were also elevated. Since age 35, recurrent electrolyte disturbances (hypokalemia and hyponatremia) and hyperuricemia developed. At age 36, he experienced a first episode of disorientation, impaired cognition, bradyphrenia, and secessus urinae. Clinical exam revealed bilateral ophthalmoparesis and dysarthria. Magnetic resonance imaging (MRI) showed subcortical hyperintensities in a nonvascular distribution, which were hyperintens on diffusion-weighted imaging (DWI) and apparent diffusion coefficient (ADC) (Figure 1). Neuropsychological testing revealed attention deficits, disability of encoding visual contents, adjustment disorder, and depression. The patient was diagnosed with suspected Bickerstaff encephalitis and questionable seizures. Following these episodes, his gait became disturbed with occasional falls due to increased ataxia. At age 37, he experienced an untriggered rhabdomyolysis. Muscle biopsy from the deltoid muscle suggested muscular dystrophy as only fat but no muscle tissue was detected (Table 2). At age 38, he underwent a second muscle biopsy from the left lateral vastus muscle which showed cytochrome-C-oxidase (COX)-negative fibers (Figure 2(a)), glycogen depositions (Figure 2(b)), fiber splitting, and ragged-red fibers (Figure 2(c)). Electron microscopy showed abnormally shaped and structured mitochondria, megaconia, abnormal glycogen and lipid depositions, and dark bodies (Figures 2(d) and 2(e)). At age 44, migraine developed. At age 47, he underwent cataract surgery bilaterally (Table 1). At age 49, diabetes was diagnosed. Transthoracic echocardiography was normal except for mild enlargement of the ascending aorta (Figure 3).

The family history was positive for hypotonia (mother), diabetes (father, brother of father, grandmother from the father's side, grand-grandfather from the father's side), carcinoma (father), and stroke (father). Neurological exam at age 49, revealed short stature, microcephaly, facial dysmorphism, myopathic face, ophthalmoparesis, pupils nonreactive to light, anisocoria (left: 5 mm, right: 3 mm, unrounded), divergence of bulbs, ophthalmoparesis (abduction right bulb 60° abduction left bulb: 15° vertical movements impossible), bilateral ptosis (right > left), reduced corneal reflexes, hypoacusis requiring bilateral hearing devices, dysarthria, dropped head, weakness for right elbow extension and flexion, reduced tendon reflexes on the upper limbs, ataxia and brady-dysdiadochokinesia on the upper limbs, marked wasting of the thighs, ataxia on the lower limbs, truncal ataxia, and ataxic stance, and gait. Gnome calves bilaterally, which were described at age 38 were no longer present. The last medication included indapamide, furosemide/spironolactone, simvastatin, NaCl, KCl, coenzyme-Q (60 mg/d), and vitamin-D.

## 3. Discussion

The presented patient is interesting in several aspects. Though clinical manifestations of KSS developed already in early infancy and childhood, the majority of the typical phenotypic features (CPEO, pigmentary retinopathy, elevated CSF protein, and cardiac conduction defects) and hepatopathy were recognised not earlier than in adulthood. Possibly, the typical features of KSS required for establishing the diagnosis [1, 2] were present already earlier; however, they were not recognied before age 30. KSS was suspected for the first time at age 39. An argument in favor of the diagnosis of KSS is that the mother was not affected suggesting that there was no transmission of a pathogenic mtDNA variant but rather spontaneous occurrence of a single mtDNA deletion, which is the cause in 96% of cases [3].

Another interesting point is the phenotype. Though the patient fulfilled the diagnostic criteria for KSS, he additionally presented with a plethora of manifestations, of which some have been reported earlier in KSS. In addition to the features required for diagnosing KSS, the patient presented with the known features of diabetes [4], hypocorticism (hyponatremia) [5], short stature [6], white matter lesions [7], hypoacusis [1], migraine [8], hepatopathy [9], steatosis [10], and cataract [11]. Features so far unreported in KSS include congenital anisocoria, severe caries, liver cysts, pituitary enlargement, desquamation of hands and feet, bone chondroma, aortic ectasia, dermoidal cyst, and polyposis of nasal sinuses. Whether all these features are truly attributable to the underlying metabolic defect remains speculative and requires further investigations, but from other MIDs it is well known that such features can be part of the phenotype [12]. The reason for the high number of novel phenotypic features could be that previous cases were not thoroughly investigated or that the genetic cause of the present patient was different from that of the previous cases.

Concerning the recurrent deteriorations of the phenotype during infections or spontaneously, it cannot be excluded that these conditions were in fact seizures or stroke-like episodes (SLEs), which remained unrecognized and recovered spontaneously. At least 4 of these episodes were documented. An argument for SLEs is that MRI at the first episode at age 36 showed subcortical T2-hyperintensities, which were also hyperintense on DWI and ADC and not confined to a vascular territory, suggesting a vasogenic edema. Whether arterial hypertension should be regarded as a manifestation of the MID is questionable but there are indications from Chinese studies that arterial hypertension can be in fact a phenotypic feature of an MID [4].

Limitations of the study were that the diagnosis was not confirmed by genetic investigations, that the mother was not investigated for the presence of a MID, and that no biochemical investigations of the muscle homogenate were carried out.

In conclusion, this case shows that KSS is indeed a multisystem disease affecting all types of tissues and that the phenotypic presentation may be broader than so far anticipated.

## Figures and Tables

**Table 1:** Disease trajectory of the presented patient over 49 years of age.

Onset (age)	Manifestation	Therapy
Intrauterine	Premature birth (7 weeks earlier)	None
Birth	Congenital anisocoria	None
Infancy	Developmental delay	None
Infancy	Hypoacusis	Hearing devices since age 39
Childhood	Downslowing, chronic fatigue	None
Childhood	Sicca syndrome + recurrent conjunctivitis	Eye drops
Childhood	Recurrent respiratory infections	Antibiotics
Childhood	Recurrent herpetic infections, stomatitis	Virostatics
5 y	Painless desquamation of hands and feet	None
6 y	Ophthalmoparesis (first recognised)	None
7 y	Tetra-ataxia	None
18 y	Severe caries	Full prosthesis
27 y	Right-sided inguinal hernia	Bassini surgery
29 y	Sacral dermoidal cyst	Surgery
30 y	Bifascicular block^*∗*^	None
30 y	Erythema nodosa of feet	Steroids
35 y	Hyper-CKemia	None
35 y	Hyperuricemia	Diet
35 y	Hyponatremia, hypokalemia	NaCl, KCl
36 y	Somnolence, infection, suspected BE, SLE	Antibiotics, virostatics, endobolin, steroids
36 y	Suspected SLE	None
36 y	Elevated CSF protein	None
36 y	Dysarthria	None
36 y	Polyposis of nasal sinuses	None
36 y	Gait disturbance, falls	None
36 y	Neuropsychological deficits	Training
37 y	Arterial hypertension	Antihypertensives
37 y	Hepatopathy	None
37 y	Liver cysts	None
37 y	Rhabdomyolysis (CK: 2673 U/L)	None
38 y	Chondroma/fibroma of the right femur	None
38 y	Steatosis hepatis	None
44 y	Suspected SLE, Na↓, K↓, migraine	Analgesics
45 y	Fall, rib fracture, pneumothorax	Drainage
45 y	Diabetes	Antidiabetics
47 y	Cataract bilaterally	Surgery
49 y	Aortic ectasia	None

^*∗*^Left anterior hemiblock plus right bundle branch block, SLE: stroke-like episode, nr: not relevant, CSF: cerebrospinal fluid, BE: Bickerstaff encephalitis.

**Table 2:** Crucial investigations carried out during the disease trajectory.

Date/age	Investigation	Result
2000, 30 years	Echocardiography	“Pulmonary insufficiency”
1.1.06	Lumbar puncture	Protein 167 mg/dl, otherwise normal
3.1.06	EEG	Normal
3.1.06	Lumbar puncture	Protein 107 mg/dl, otherwise normal
5.1.06, 35 years	Nerve conduction studies	Normal
4.1.06, 36 years	Cerebral MRI	T2-hyperintensities, subcortical, mesencephalon, DWI, ADC hyperintense enlarged pituitary gland, sinusoidal polyposis
12.1.06	Neuropsychological testing	Attention deficit, disability of encoding visual contents, adjustment disorder, and depression
13.1.06	Cerebral MRI	Unchanged to previously
17.1.06	Lumbar puncture	Protein 152 mg/dl, otherwise normal
1/2006	Ophthalmologic investigation	Strabism, fundus tabulates, AM atrophy, papillary excavation
24.2.06	Cerebral MRI	Band-like T2-, DWI-, ADC-hyperintensities in the ML and mesencephalon bilaterally
2.1.06	Abdominal ultrasound	Normal
1/06	Lumbar puncture	Protein 167 mg/dl
16.12.06	Ophthalmologic investigation	Pigmentary epithelium shifts, retinal dystrophy, rod/cone degeneration (RP suspected)
8/07	Muscle biopsy (deltoid)	Fat, no muscle
12.9.07	CK	2673 U/L (rhabdomyolysis)
10.8.07	Myoglobin	152 ng/ml (n, 19–92 ng/ml)
2.8.07	X-ray of the lungs	Normal width of aorta
6.8.07	Echocardiography	Normal
8.8.07	Liver enzymes	Elevated
9.8.07	Abdominal CT	Multiple liver cysts
13.8.07	Muscle biopsy	No muscle tissue, only fat
13.2.08	Muscle MRI	Chondroma right femur
19.2.08, 38 years	Muscle biopsy (left lateral vastus)	Indicates mitochondrial myopathy
15.12.08	Cerebral MRI	Cerebellar atrophy
19.12.08	Needle electromyography	Increased polyphasia exclusively

WMLs: white matter lesions, RP: retinitis pigmentosa.
